# Racial and Ethnic Variations in Preventive Dental Care Utilization among Middle-Aged and Older Americans, 1999–2008

**DOI:** 10.3389/fpubh.2013.00065

**Published:** 2013-12-17

**Authors:** Bei Wu, Jersey Liang, Huabin Luo, Robert Furter

**Affiliations:** ^1^School of Nursing, Global Health Institute, Duke University, Durham, NC, USA; ^2^School of Public Health, University of Michigan, Ann Arbor, MI, USA; ^3^East Carolina University, Greenville, NC, USA; ^4^University of North Carolina at Greensboro, Greensboro, NC, USA

**Keywords:** racial disparity, dental care utilization, older adults, middle-aged, trends

## Abstract

**Objective:** This study examined recent trends of preventive dental care utilization among Americans aged 50 and above, focusing on variations across racial and ethnic groups including Whites, Blacks, Hispanics, American Indians/Alaska Natives, and Asians.

**Methods:** Self-reported information on oral health behaviors was collected from 644,635 participants in the Behavioral Risk Factor Surveillance System between 1999 and 2008.

**Results:** Despite a significant upward trend of frequency of dental cleaning from 1999 to 2008 (OR = 1.02), in 2008 still only 56–77% of any ethnic or racial group reported having had a dental cleaning in the previous 12 months. Relative to Whites, Blacks (OR = 0.65) were less likely to have a dental cleaning in the previous 12 months. These variations persisted even when SES, health conditions, health behaviors, and number of permanent teeth were controlled. In contrast, Hispanics, Asians, and American Indians/Alaskan Natives did not differ from Whites in dental cleanings.

**Discussion:** This is the first study to provide national estimates of the frequency of dental cleaning and associated trends over time for five major ethnic groups aged 50 and above in the U.S. simultaneously. Our findings suggest that public health programs with an emphasis on educating middle-aged and older minority populations on the benefits of oral health could have a large impact, as there is much room for improvement. Given the importance of oral health and a population that is rapidly becoming older and more diverse, the need for improved dental care utilization is significant.

## Introduction

Among the many health care issues that middle-aged and older adults face, care of one’s teeth is one that may have serious repercussions if overlooked. Neglect of oral care may lead to tooth decay, pain, tooth loss, and inflammation. More importantly, untreated or delayed treatment of oral diseases affects nutritional status, causes oral pain and discomfort, and ultimately affects individuals’ quality of life (1). Over the past several decades, dental care utilization has risen considerably (2, 3). Specifically, national surveys have reported that the proportion of respondents who had seen a dentist in the past year rose from an estimated 53.9 in 1983 to 75.5 in 1997, for adults 25 and older, with women reporting higher rates than men throughout the study period (3). In the past, rates of dental care use also differed across racial/ethnic groups. Whites reported consistently higher rates than Blacks and Hispanics, with Hispanics usually falling somewhere between the White and Black rates (2, 3). Based on data from three large national surveys, the proportions of Whites seeking dental care increased from 46.89 to 47.84%, while the proportion of Blacks increased from 23.44 to 26.73%. The proportion for Hispanics stayed approximately the same over the time period (29.86% in 1996). This study also found an increase in the proportion of individuals over the age of 65 that had seen a dentist in the past year between 1977 and 1996 (29.88–41.33%) (2). While prior reports presented findings regarding utilization rates of individuals in earlier time periods; no up-to-date statistics on whether rates of utilization meet the annual dental checkups recommended by the US Department of Health and Human Services (4). Further, most of these prior studies only reported cursory descriptive results, and have not included formal statistical tests by adjusting for population heterogeneity that could potentially contribute to the impact of race/ethnicity on dental care utilization.

Limited research is available on dental care utilization among American Indians/Alaskan Natives; however, the available research on this group has focused on the lack of access American Indians have to health care. Many American Indians suffer from a lack of access to dental care as a result of under-staffed medical centers, excessive estimated wait times at medical centers, and/or distances that are too long to travel for such medical services (5). Such factors clearly have a negative impact on American Indians opportunity and propensity to utilize dental care.

Similarly, few studies are available on dental care use among middle age and older Asian Americans in nationally representative samples. Using the National Health Interview Survey (NHIS) (1997–2000) data, one descriptive study reported that 58% of the Asians aged 65 and above had visited a dentist in the previous year, slightly higher than the rate in Whites (56.6%); while the rate for Asians aged 45–64 was 66.1%, slightly lower than their white counterparts (67.6%) (6). While the NHIS study was administered in English to the Asian participants, using a convenient sample, another study conducted among Chinese American elders in their native language found that only 47% of the participants aged 60 and above reported having a dental visit within the previous year (7).

These prior studies examined changes in dental care use in regard to certain racial/ethnic groups, primarily Whites, Blacks, and Hispanics, with the majority excluding groups such as Asians and American Indians/Alaskan Natives. While many of these studies looked at a range of age groups, few targeted adults over age 50. Those studies that included individuals over age 50 presented results with the aim of contrasting with other age groups (2, 8). The number of middle-aged and older adults has increased significantly for the past several decades (9) and has become increasingly diverse (10). Studies have shown that there is an increasing number of middle-aged and older adults who are retaining their natural teeth (11–13). These findings highlight the importance of dental care use for this segment of the population.

This study is unique in that it studies dental care use among Asian and Native Americans in comparison with other major racial/ethnic groups using a nationally representative survey. Overall, Asian Americans may have a higher use of preventive dental care than other minority populations in the U.S. On the other hand, American Indians have less access to dental care which would affect their use of preventive care. However, no studies have empirically examined the dental care utilization among Asian and Native Americans using a national representative survey. It is unknown whether the use of dental care would be explained by other confounding factors for these population segments.

To guide the analysis, this study offers the following hypotheses.

First, preventive dental care use among middle-aged and older Americans increased between 1999 and 2008 (H_1_) (2, 3).

Second, significant race/ethnic differences exist in the trend of preventive dental care utilization. In particular, preventive oral care use is highest among white Americans and the lowest among black Americans and American Indians, with Hispanic and Asian Americans somewhere in between (H_2_) (2, 5, 6).

Third, on the basis of the Behavioral Model of the Health Services (14, 15), this study hypothesizes that racial/ethnic variations in preventive dental care utilization are partially accounted for by heterogeneity in predisposing factors (e.g., demographic characteristics and health behavior), enabling factors (e.g., education, income), and needs (e.g., health conditions). With regard to predisposing factors, females and married individuals are more likely to seek regular dental care (8, 13). Evidence has consistently shown that smoking is a risk factor for oral health (16, 17) and individuals who smoke use dental services less often (7, 18). Compared to those who abstain from drinking alcohol, one study found that moderate drinkers had a higher use of dental care (19). One study has shown that the relationship between number of teeth and body mass index (BMI) is not linear. Individuals who were either underweight or obese were more likely to have fewer teeth compared with those who were normal weight (20). Enabling characteristics are the personal resources available to an individual that enable or impede the use of health services. Studies found that individuals with higher levels of education and income are more likely to use dental services (8, 21–23). A growing body of literature suggests that oral health and dental care use are associated with diabetes, cardiovascular diseases, and respiratory diseases (24–28). Individuals with a higher number of natural teeth are more likely to seek preventive dental care (11, 19). Need exists when an individual utilizes healthcare services based on their illness (29). Literature also suggests that oral health and dental care use are associated with diabetes, cardiovascular diseases, and respiratory diseases (24–28).

## Materials and Methods

### Data sources and sample

This study used the Behavioral Risk Factor Surveillance System (BRFSS), which is a telephone health survey developed by the National Center for Statistics and Prevention and administered yearly. The main goal of the BRFSS is to track health conditions and their risk factors in the United States. For this study, five waves of BRFSS data collected between 1999 and 2008 were aggregated. Eligible respondents were aged 50 and above, and completed the survey item regarding their last dental cleaning. Given that individuals who have lost all their natural teeth are less likely to use preventive dental care (i.e., dental cleaning), and thus, may have a very different pattern of dental care use (30), only respondents who have not lost all of their natural teeth were included in this study. The sample included 8,808 American Indians/Alaska Natives, 10,043 Asians, 35,371 Hispanics, 51,744 Blacks, and 640,063 non-Hispanic Whites.

### Measures

#### Dependent variable

The dependent variable for this study included responses to a survey question, “How long has it been since you had your teeth cleaned by a dentist or dental hygienist?” The variable was coded dichotomously as 1 if the respondent had a dental cleaning within the past year, and 0 otherwise. Respondents’ last dental visit was also assessed; the results showed that there was a high correlation between dental visits and dental cleanings (*r* = 0.82) and the results from parallel multivariate analysis were also similar. Given the reasons for the last dental visit were unknown (e.g., can be for either preventive care or treatment), only the findings on the dental cleaning outcome are reported. Self-reported dental cleaning is a commonly used and valid measure of preventive dental care use (31).

#### Independent variables

Ethnicity was measured using dichotomous variables for American Indians/Alaskan Natives, Asian Americans, Blacks, Hispanics, and non-Hispanic Whites. The years were coded from one to five, corresponding to the years with relevant dental questions included in the years of the survey (i.e., 1999, 2002, 2004, 2006, and 2008, respectively).

#### Covariates

The socio-demographic variables included in the analyses were age (1 = 50–64 years, 2 = 65–74, 3 = 75, or over), gender (1 = female, 0 = male), marital status (1 = married, 0 = not married), education (1 = at least a high school education, 0 = less than a high school education), income (1 = less than $10,000, 2 = 10,000 − 14,999, 3 = 15,000 − 19,999, 4 = 20,000 − 24,999, 5 = 25,000 − 34,999, 6 = 35,000 to 49,999, 7 = 50,000 − 74,999, 8 = 75,000 and above), and health insurance (1 = having health care coverage, 0 = otherwise).

Health-related factors were also addressed in the analyses. The presence of several medical conditions was assessed by asking the respondents whether they had ever been told by a doctor that they had diabetes, a heart attack, heart disease, stroke, or high blood pressure. These variables were evaluated dichotomously. BMI was calculated using self-reported height and weight, then coded appropriately into one of four categories (underweight = <18.5; normal weight = 18.5–24.9; overweight = 25.0–29.9; and obese = 30 or greater). Other health-related behaviors and factors were also addressed, such as smoking and alcohol consumption. Smoking was coded dichotomously based on whether the respondent had smoked 100 cigarettes in their lifetime. Alcohol consumption was coded as the number of days in the last 30 days the respondent had consumed alcohol. Oral health was considered in the analyses by the number of natural teeth the respondent had removed (1 = more than five natural teeth removed but not all, 2 = one to five teeth removed, 3 = no natural teeth removed).

### Data analysis

SAS 9.2 was used for the analyses in this study. The Survey Means procedure was used to examine the weighted means for each variable within each race/ethnicity and year, looking specifically at sex, education, and age. Since the dental cleaning variable is dichotomous, the predicted probability of the use rate was calculated using logistic regression adjusting for time, age, race/ethnicity, gender, and level of education. To estimate the rate of dental cleaning, the value of the probability of dental cleaning was summed for all respondents within each racial/ethnic group per year and the sum total was divided by the number of participants in the corresponding group.

Weighted logistic regressions were performed with dental cleaning as the outcome variable using the computed weight assigned to each individual. The first model consisted of time and race/ethnicity as predictor variables. The second model included age, sex, marital status, education, income, and insurance. The third model added health conditions, such as diabetes, heart disease, and BMI, and health-related behaviors, such as smoking and alcohol consumption. The fourth step created a model that incorporated the number of natural teeth the respondent had removed. Product terms between each of the race/ethnicity variables and time were tested and added in the fifth step. All these product terms were not significant, thus, the results from step 5 were not presented in the study. Because of the large sample, statistical significance was defined as *p* < 0.01.

## Results

### Sample characteristics

Many sample characteristics were significantly different across the racial/ethnic groups (Table 1). Blacks, Hispanics, and American Indians/Alaskan Natives had a lower proportion of respondents that were married in comparison to the White reference group, with Asians being the only group with a significantly higher proportion than the reference group. Blacks, Hispanics, Asians, and American Indians/Alaskan Natives were found to have a significantly lower proportion of respondents that had at least a high school education and health insurance coverage. American Indians/Alaskan Natives were the only ethnic group to have a higher proportion of respondents than Whites that have smoked at least 100 cigarettes, with the other groups found to be significantly lower.

**Table 1:** **Sample characteristics by race/ethnicity**.

Variables	Descriptive (unit = %)				
	White	Black	Hispanic	Asian	AIAN^a^
	*n* = 554,775	*n* = 42,737	*n* = 30,833	*n* = 9328	*n* = 6962
	Mean/% (SE)	Mean/% (SE)	Mean/% (SE)	Mean/% (SE)	Mean/% (SE)
Age	63.70 (0.03)	61.41 (0.09)	61.13 (0.12)	60.94 (0.23)	60.55 (0.26)
Female	53.85	56.61	53.45^NS^	50.87	48.04
Married	70.13	47.64	66.55	73.04	65.49
Education (at least a high school education)	93.05	79.92	60.62	78.70	74.28
Insurance	94.59	86.12	80.65	87.68	81.84
Diabetes	11.35	22.72	22.14	16.86	20.29
Heart attack	7.81	7.58^NS^	7.83^NS^	6.05	12.93
Coronary heart disease	8.82	6.95	7.73	6.84	10.10
Stroke	4.33	6.81	4.33^NS^	3.32	8.15
High blood pressure	40.65	58.55	41.92^NS^	39.52^NS^	38.81^NS^
Smoked at least 100 cigarettes	50.76	48.74	40.64	34.27	54.74
Days consumed alcohol in the last 30 days	5.78 (0.03)	2.55 (0.09)	2.63 (0.10)	2.73 (0.24)	3.9 (0.37)
Underweight	5.56	5.54^NS^	10.80	7.17	6.90
Normal weight	33.57	22.33	24.26	41.45	27.53
Overweight	38.81	37.93	37.86	37.33	37.04
Obese	22.07	34.20	27.08	14.04	28.53
No teeth removed	37.12	16.85	30.00	35.97^NS^	28.44
1–5 Teeth removed	39.56	42.34	45.94	42.24	39.44^NS^
5+ Teeth removed	23.32	40.81	24.06	21.78	32.11

*NS, not significant at *p*-value 0.01 (in comparison to White as a reference group)*.

^a^ American Indian/Native Alaskan

### Last dental cleaning across racial/ethnic groups

Over the past 10 years, the proportion of respondents who had a dental cleaning in the preceding year increased across every ethnic group. Results from the weighted descriptive analysis revealed that Asians had the highest increase in the observed rate of dental cleanings during the period of 1999 and 2008 and also had the highest proportion of dental cleanings in 2008 (76.69%), followed by Whites (76.18%), Hispanics (62.33%), American Indians/Alaskan Natives (61.85%), and Blacks (56.52%) (Table 2). The predicted rates showed a similar rank order except for Asians and Whites. White Americans had the highest predicted rate of dental cleaning (76.13%), followed by Asians (74.97%). Overall, individuals with more years of education had a higher rate of dental cleaning in the previous year. Females, White females in particular, had a higher rate of dental cleaning than their male counterparts. While younger participants had an overall high observed rate of dental cleaning for blacks, the pattern of dental cleaning across age groups was less clear for other racial/ethnic groups.

**Table 2:** **Trend. of last dental cleaning by racial/ethnic groups (1999–2008) (weighted)**.

Year	Sample size (*N*)	Observed (%)	Predicted (%)	Gender (%)		Education (%)		Age groups (%)		
				Male	Female	<High school	≥High school	50–64	65–74	75+
**White**	**554,775**									
1999	43,371	75.34	75.06	73.71	76.70***	52.00	77.96***	74.82	76.38*	75.62
2002	76,695	75.52	75.71	73.47	77.27***	48.65	77.74***	76.09	75.01*	74.18***
2004	106,793	75.91	75.97	74.15	77.42***	48.55	77.72***	75.88	76.31	75.60
2006	142,313	75.61	76.01	73.76	77.21***	45.77	77.47***	75.36	76.20*	75.88
2008	185,603	76.18	76.13	74.35	77.77***	47.26	77.73***	75.91	77.07***	76.24
**Black**	**42,737**									
1999	2990	55.88	52.67	54.55	56.91	38.35	62.50***	60.98	47.40***	37.56***
2002	5359	54.82	53.71	52.87	56.20	34.91	60.32***	56.87	51.15*	46.92***
2004	8424	54.11	54.56	51.60	55.94***	35.20	58.52***	56.14	51.30*	46.50***
2006	11,292	52.73	54.89	50.64	54.45***	28.48	58.20***	54.37	48.16***	49.78*
2008	14,672	56.52	55.25	55.34	57.47	34.35	60.27***	57.18	56.07	52.97*
**Hispanic**	**30,833**									
1999	282	61.26	66.29	59.36	63.04	31.23	69.88***	62.75	68.03	45.64
2002	4750	59.75	61.93	59.01	60.39	44.67	70.30***	60.14	62.88	50.69***
2004	6460	60.54	61.53	58.70	62.07*	51.03	66.95***	60.65	60.49	59.95
2006	8640	58.23	61.89	54.80	61.28***	43.04	67.80***	58.63	59.29	54.38
2008	10,701	62.33	62.01	59.51	64.85***	52.79	67.78***	61.58	64.89*	63.23
**Asian**	**9328**									
1999	2122	62.84	67.78	58.62	66.33**	50.43	72.19***	62.56	66.76	54.16
2002	1608	76.67	74.89	71.31	82.02***	73.70	77.06	77.43	74.31	75.11
2004	699	77.22	75.98	78.38	75.98	64.49	78.24	78.08	75.12	73.31
2006	2127	77.72	75.40	76.89	78.58	44.02	78.62***	79.46	70.11*	78.42
2008	2772	76.69	74.97	76.91	76.42	59.90	79.02***	78.38	73.15	71.18*
**AIAN^a^**	**6962**									
1999	708	57.49	54.16	59.40	55.50	45.50	72.53***	57.86	52.99	62.32
2002	991	65.77	60.99	61.21	71.38**	46.92	69.06***	67.21	66.19	57.63
2004	1382	57.53	61.46	52.68	62.41**	39.22	61.28***	55.85	66.40	61.04
2006	1661	53.79	61.50	51.42	56.61	35.65	57.04***	51.32	66.34***	53.12
2008	2220	61.85	61.92	61.89	61.81	37.64	64.79***	62.69	63.84	53.89*

*The predicted probability of the use rate was calculated using logistic regression adjusting for time, age, race/ethnicity, gender, and level of education*.

*^a^ American Indian/Alaskan Native*.

*0.01 Level of significance.

**0.001 Level of significance.

***0.0001 Level of significance.

### Last dental cleaning logistic regression

The proportion of individuals that had a dental cleaning in the previous year increased overall from 1999 to 2008 (OR = 1.016, 99% CI: 1.005, 1.027) (Table 3, Model 1). In comparison to the White reference group, Black (OR = 0.388, 99% CI: 0.368, 0.409), Hispanic (OR = 0.483, 99% CI: 0.449, 0.519), Asian (OR = 0.806, 99% CI: 0.699, 0.899), and American Indian/Alaskan Native (OR = 0.465, 99% CI: 0.399, 0.543) respondents were less likely to have had a dental cleaning in the last year.

**Table 3:** **Logistic regression on last dental cleaning (dichotomous)**.

Variables	Model 1		Model 2		Model 3		Model 4	
	Odds ratio	99% Confidence interval	Odds ratio	99% Confidence interval	Odds ratio	99% Confidence interval	Odds ratio	99% Confidence interval
Time	1.016	(1.005, 1.027)***	0.952	(0.940, 0.964)***	0.973	(0.791, 1.197)	0.982	(0.794, 1.214)
Black	0.388	(0.368, 0.409***	0.640	(0.599, 0.684)***	0.630	(0.470, 0.845)***	0.654	(0.483, 0.885)***
Hispanic	0.483	(0.449, 0.519)***	1.209	(1.1023, 1.325)***	0.950	(0.469, 1.926)	0.891	(0.453, 1.756)
Asian	0.806	(0.699, 0.930)***	1.261	(1.057, 1.506)*	0.679	(0.116, 3.97)	0.725	(0.122, 4.310)
AIAN^a^	0.465	(0.399, 0.543)***	0.871	(0.722, 1.049)	0.905	(0.493, 1.663)	0.885	(0.473, 1.655)
Age 65–74			1.251	(1.194, 1.311)***	1.320	(0.983, 1.771)	1.463	(1.081, 1.980)**
Age 75+			1.438	(1.368, 1.512)***	1.241	(0.898, 1.714)	1.362	(0.984, 1.886)
Female			1.480	(1.428, 1.535)***	1.320	(1.046, 1.665)*	1.331	(1.050, 1.687)*
Education			1.767	(1.656, 1.885)***	2.029	(1.484, 2.773)***	1.989	(1.439, 2.745)***
Married			1.039	(0.998, 1.081)	0.968	(0.763, 1.230)	0.964	(0.755, 1.231)
Income			1.358	(1.344, 1.373)***	1.370	(1.281, 1.465)***	1.326	(1.238, 1.420)***
Insurance			2.301	(2.145, 2.467)***	2.339	(1.633, 3.349)***	2.383	(1.652, 3.438)***
Diabetes					0.849	(0.634, 1.136)	0.843	(0.624, 1.138)
Heart attack					0.723	(0.470, 1.112)	0.708	(0.459, 1.092)
Heart disease					0.859	(0.568, 1.301)	0.844	(0.554, 1.286)
Stroke					0.890	(0.544, 1.456)	0.886	(0.543, 1.446)
High blood pressure					1.076	(0.867, 1.336)	1.099	(0.882, 1.371)
Smoking					0.712	(0.575, 0.882)***	0.800	(0.641, 0.997)*
Alcohol					0.998	(0.979, 1.018)	0.995	(0.975, 1.015)
Underweight					0.749	(0.458, 1.225)	0.776	(0.465, 1.294)
Overweight					0.980	(0.745, 1.289)	1.033	(0.783, 1.363)
Obese					0.796	(0.587, 1.079)	0.811	(0.596, 1.103)
Permanent teeth							1.507	(1.300, 1.747)***

*^a^ American Indian/Alaskan Native*.

*0.01 Level of significance.

**0.001 Level of significance.

***0.0001 Level of significance.

After controlling for demographic characteristics, medical conditions, health behaviors, and the number of permanent teeth, compared with White respondents, Black respondents (OR = 0.654, 99% CI: 0.483, 0.885) were the only racial/ethnic group significantly less likely to have had a dental cleaning in the past year. Older individuals who were female, had higher levels of education and income, had insurance coverage, and had a higher number of natural teeth were more likely to have a dental cleaning in the previous year. On the other hand, individuals who had heart attack and smoked were less likely to have a dental cleaning in the preceding year.

## Discussion

This is the first study to provide national estimates for dental cleaning and associated trends over time for five major ethnic groups aged 50 and above in the U.S. simultaneously: American Indian/Alaskan Native, Asian Americans, Blacks, Hispanics, and non-Hispanic Whites. Most previous studies on trend of dental care use have focused on children and young adults, with just a small number on aged 50 and above (2, 8). Given the increasing number of middle-aged and older adults in the U.S who need dental care, research is needed to examine the trend of dental care use and factors related to dental care use in this segment of the population.

The results support the first hypothesis that use of preventive dental care continued to increase between the period of 1999 and 2008, extending beyond the previously reported increase for the periods of the 1970s and 1990s (2, 3). However, the increase in dental care was largely explained by heterogeneity in socio-demographic and health attributes over time. The second hypothesis (H_2_) was also supported by the study findings. Blacks and American Indians had the lower predicted rate of preventive dental care in the U.S. The study also supports the third hypothesis (H_3_) that racial/ethnic variations in preventive dental care utilization are partially accounted for by population heterogeneity. There were differences in dental care use over time between White and minority groups. However, these differences in dental cleaning over time between White Americans and other minority groups, except Blacks, can be explained by socio-demographic and health characteristics. Differences between Blacks and Whites persisted even when population heterogeneity was adjusted. The results from product terms suggest that there were no significant trend differences across racial/ethnic groups with regarding to preventive dental care over the past 10 years.

This study found that health insurance, education, income, smoking, and the number of natural teeth were among the covariates that were associated with dental cleanings. The steadily rising level of education over the past 10 years may partially contribute to the increase of preventive dental care use over the same time period (32). Education is a key to promoting healthy behaviors (21). Individuals with a higher level of education may have better knowledge of oral health practices, and the risks associated with dental care neglect. Health insurance and income could reflect an enabling issue and be indicative of improved access to dental care with greater resources.

Having a dental cleaning in the past year could reflect a positive health seeking behavior. Specifically, smoking was found to have a negative effect on the likelihood of a dental cleaning, indicating less of a positive health seeking attitude for smokers. This finding is of substantial importance, as smokers’ oral health is not only affected by the use of tobacco, but also by the neglect of under-utilizing dental care. Use of tobacco and dental care may be inversely related. The recent decreasing use of tobacco and the increase use of preventive dental care are likely associated with greater awareness of health issues in the U.S. Women were more likely to have a cleaning than men. The results confirm the findings from previous studies (8, 13) and are also consistent with the literature on preventive medical care (33, 34). Some researchers speculate that women’s more frequent preventive health services use relates to their acceptance of help seeking, compliance with treatment regimens, and willingness to adopt the sick-role (33–35). Thus, targeting health attitudes and behaviors that vary with gender might be the most effective strategies for producing changes in preventive dental care.

This study expanded upon previous studies by adjusting potential confounding factors and found improvements in dental care utilization were not equally distributed across the ethnic groups. Compared to Whites, Blacks were less likely to utilize dental care; however, the magnitude of the disparity decreased when controlling for socioeconomic status, medical conditions, health behaviors, and the presence of permanent teeth. These findings suggest the presence of other systematic differences in factors between the Black and White groups in regard to receiving dental cleanings, and issues such as quality of dental care and priority of use of preventive health services, may help to explain these findings.

The current study goes beyond that of prior research by comparing Asians to Whites in addition to other ethnic groups. After controlling for covariates, the disparities in dental care utilization between Asians and Whites are no longer present. However, the results are likely to be inconclusive given the caveat of the BRFSS sample excluding non-English speaking individuals in the study. The rates of dental cleaning for Asian Americans in our study were higher than the rate reported from Wu et al.’s (7) study conducted among Chinese American elders aged 60 and older in 2000. One major reason for the rate discrepancy is likely due to the differences in data collection: the previous one was conducted in Chinese and the BRFSS survey was conducted in English. According to the 2009 U.S. Census data, 32% of Asian Americans have limited English proficiency. Lack of English proficiency is most common for Vietnamese (51%), Koreans (41%), and Chinese (42%) (36). Given the fact that national surveys, such as BRFSS, do not interview Asian Americans in their native language, the surveys are most likely to exclude individuals who have limited English language skill, and consequently, by large, excluding those with lower SES. Individuals with different levels of SES are likely to have different pattern of preventive dental care. The Asian population in the United States warrants researchers’ attention as it is growing at a faster rate than any other ethnic groups, and is exceptionally diverse (37). Noting the heterogeneous nature of the Asian American population, past research has examined how subgroups within the Asian population compare to each other in regard to dental care utilization (6), and future research could be conducted to show how these subgroups compare to a White reference group and other minority groups.

This study also provides new knowledge on dental care use among American Indians. Prior research has identified the problems many American Indians/Alaskan Natives may face in seeking medical care (5). Other research has documented that American Indians/Alaskan Natives utilize general healthcare at lower rates in comparison to Whites; however, like the findings of the current study, these disparities are no longer present after adjusting for other factors, such as education, income, health insurance, and health behaviors (38). The findings suggest that much of the disparity of preventive dental care for American Indians could be explained by their low socioeconomic status and unhealthy behavior such as smoking. Many of the American Indians reside on reservations. Looking past the dental care utilization of American Indians and focusing on the quality of care American Indians receive through Indian health services (IHS); reports have indicated that American Indians do not receive adequate dental care. For reasons such as salary levels, length of commitment, more attractive employment opportunities elsewhere, and other factors, recruitment and retention of dental health care professionals has become a continuing and worsening problem for IHS. Currently, 32% of full-time dental positions in IHS are vacant, an all-time high (4). For this reason, current SES or contemporary barriers to dental care may not fully capture the experience and problems American Indians/Alaskan Natives face in securing consistent and adequate care, and lower utilization may indicate an accumulation of disadvantages over time.

While some evidence suggests the association between oral health and systemic health (39), our study did not find a significant association between dental care use and many chronic conditions. It is possible some of the association is explained by other factors such as socioeconomic status, health behaviors (e.g., smoking) that are included in the study. Further study is warranted to further examine the complex relationship between oral health, dental care, and systemic diseases.

### Study limitations

The BRFSS survey data has some inherent limitations. As a telephone survey, individuals without telephones are naturally not represented. Also, information in the survey is self-reported, leaving the accuracy of when an interviewee’s last dental cleaning occurred subject to their best recollection. While the sample size of certain demographics and minority groups in some study years was relatively small in comparison to other groups, survey weights were used to adjust for this in the analyses. The survey data also lacked information on dental insurance and oral hygiene behaviors that could affect our measures of utilization. Future research is needed to examine subgroups within ethnic groups given their heterogeneous nature, as well as to evaluate the specific causes behind differences in dental care utilization past the predictors examined in this study.

## Conclusion

An overall upward trend in dental care utilization between 1999 and 2008 was observed; however there were some significant differences and variations across the five major racial/ethnic groups. Public health programs with an emphasis on educating and enabling middle-aged and older minority populations to the benefits of oral health could have a large impact, as there is much room for improvement. Given the importance of oral health and a population that is rapidly becoming older and more diverse the need for improved access to dental care is significant.

## Conflict of Interest Statement

The authors declare that the research was conducted in the absence of any commercial or financial relationships that could be construed as a potential conflict of interest. The Associate Editor Jie Hu declares that, despite being affiliated with the same institution as the author Robert Furter, the review process was handled objectively and no conflict of interest exists.
